# Treatment patterns and clinical outcomes according to PD-L1 status in >2000 patients with early-stage or metastatic triple-negative breast cancer treated in the real-world setting: VANESSA study results

**DOI:** 10.1016/j.breast.2026.104720

**Published:** 2026-02-05

**Authors:** Lazar Popovic, Romualdo Barroso-Sousa, Nagi S. El Saghir, Rebecca Dent, Sitki Tuzlali, Saad Akhtar, Elona Juozaityté, Janis Eglitis, Dinesh C. Doval, Carlos A. Castaneda, Alisan Zirtiloglu, Götz Hartleben, Regula Deurloo, Paula Toro, Iman Estaytieh, Enya Weber, João Mouta, Corrado D’Arrigo

**Affiliations:** a Department for Medical Oncology, Oncology Institute of Vojvodina, Faculty of Medicine, University of Novi Sad, Novi Sad, Serbia; b Brasilia Hospital, Rede Américas, Brasilia, Brazil; c American University of Beirut Medical Center, Beirut, Lebanon; d National Cancer Center Singapore, Duke-NUS Medical School, Singapore; e Tuzlali Pathology Laboratory, Istanbul, Turkiye; f Cancer Centre of Excellence, King Faisal Specialist Hospital and Research Centre, Riyadh, Kingdom of Saudi Arabia; g Department of Oncology and Hematology, Oncology Institute, Lithuanian University of Health Sciences, Kaunas, Lithuania; h Faculty of Medicine, University of Latvia, Riga, Latvia; i Rajiv Gandhi Cancer Institute and Research Centre, New Delhi, India; j Department for Medical Oncology, Instituto Nacional de Enfermedades Neoplásicas, Faculty of Health Sciences, Universidad Cientifica Del Sur, Lima, Peru; k Global Product Development/Medical Affairs Oncology, F. Hoffmann-La Roche Ltd, Basel, Switzerland; l Medical Affairs, Roche Pharma AG, Grenzach-Wyhlen, Germany; m Translational Medicine Oncology gRED, F. Hoffmann-La Roche Ltd, Basel, Switzerland; n Global Medical Affairs, Roche Diagnostics International AG, Rotkreuz, Switzerland; o Pharmaceuticals Division, Roche Lebanon SARL, Beirut, Lebanon; p Biometrics and Epidemiology, Roche Pharma AG, Grenzach-Wyhlen, Germany; q Global Product Development/Medical Affairs Oncology, Roche Farmacêutica Química, Amadora, Portugal; r Poundbury Cancer Institute for Personalised Medicine, Dorchester, UK

**Keywords:** Real-world, PD-L1, SP142, Triple-negative breast cancer, De novo metastatic

## Abstract

**Background:**

The prognostic effect of PD-L1 status in triple-negative breast cancer (TNBC) is uncertain and little is known about PD-L1-positive prevalence and outcomes in the real-world setting.

**Patients and methods:**

The multicentre retrospective observational VANESSA study evaluated the prevalence and impact of PD-L1-positive status in 2054 patients receiving systemic therapy for early-stage or metastatic (e/m)TNBC between 2014 and 2017. PD-L1 expression was assessed locally and centrally on archival samples. Descriptive analyses of demographic and clinicopathological characteristics, treatment patterns and clinical outcomes (extracted from patients’ medical records) according to PD-L1 status were prespecified.

**Results:**

Among 1902 patients with eTNBC, 681 (36%) received neoadjuvant chemotherapy and 1261 (66%) adjuvant chemotherapy. Demographic characteristics were generally similar regardless of PD-L1 status, but lower-risk tumour characteristics were more common in the PD-L1-positive subgroup. Invasive disease-free and overall survival were more favourable in PD-L1-positive eTNBC. In the mTNBC cohort, 120/145 (83%) patients had de novo mTNBC. Median progression-free survival on first-line treatment was 7.6 months (95% CI: 4.1–15.0) in PD-L1-positive mTNBC (n = 30) and 4.9 months (95% CI: 3.6–6.1) in PD-L1-negative mTNBC (n = 83).

**Conclusion:**

In eTNBC and mTNBC, PD-L1-positive status was associated with more favourable long-term outcomes, possibly due to tumour-intrinsic characteristics and/or the host immune response. The high proportion with de novo mTNBC may suggest enrolment bias and/or geographic variations in stage at diagnosis.

## Highlights

• The impact of PD-L1 status on TNBC prognosis and real-world outcomes is uncertain.
• The VANESSA retrospective study included 2054 patients with early/metastatic TNBC.
• Outcomes appeared more favourable in PD-L1+ than PD-L1– early and metastatic TNBC.

## 1 Background

In the era of personalised medicine, reliable and precise characterisation of tumour biology is becoming increasingly important in assessing the risk of relapse/progression, determining the optimal strategy and guiding treatment selection. In many settings, including triple-negative breast cancer (TNBC), the presence or absence of pathogenic variants or other biomarkers identifies patients likely to benefit (or not) from a specific treatment and/or provides prognostic information regardless of treatment. Clinical trials in unselected populations of patients with early-stage (e)TNBC suggest that PD-L1-positive status is associated with more favourable outcomes [[1], [2], [3], [4]]. In contrast, in locally advanced/metastatic (m)TNBC, the prognostic role of PD-L1 expression is less clear, with recent trials showing similar outcomes with chemotherapy irrespective of PD-L1 status [[5], [6], [7], [8]]. Three meta-analyses exploring the potential prognostic role of PD-L1 positivity in breast cancer have yielded contradictory findings [[9], [10], [11]]. However, conclusions are limited by a lack of discrimination between early-stage versus metastatic disease, and between PD-L1 expression on immune versus tumour cells in two of the meta-analyses. PD-L1 expression may change over time and under treatment pressure, confounding potential prognostic roles. For example, in exploratory analyses of the NeoTRIP trial evaluating neoadjuvant atezolizumab, the prevalence of PD-L1 positivity increased from 45% to 75% after one cycle of carboplatin, nab-paclitaxel and atezolizumab, but decreased from 53% to 38% in patients receiving chemotherapy alone [12]. Additional studies are needed to elucidate the potential prognostic role of PD-L1 status and to develop our understanding of PD-L1-positive prevalence and outcomes in real-world settings.

The international VANESSA study assessed the global prevalence of PD-L1-positive status in eTNBC and mTNBC using the VENTANA PD-L1 (SP142) Assay (Roche Diagnostics) in real-world clinical practice. Here we report exploratory analyses relating to patient and disease characteristics potentially affecting detection of PD-L1-positive status and clinical outcomes.

## 2 Patients and methods

### 2.1 Study design

The design of the international observational secondary data-use VANESSA study has been described elsewhere [13]. Briefly, the study enrolled patients treated with systemic therapy for eTNBC or mTNBC newly diagnosed between January 1, 2014 and December 31, 2017. TNBC status was assessed locally according to ASCO/CAP guidelines [[14], [15], [16]]. Submission of good-quality archival formalin-fixed paraffin-embedded tumour tissue for PD-L1 testing was mandatory. PD-L1 status was assessed locally and at a central laboratory by pathologists trained and certified specifically for TNBC. PD-L1-positive status was defined as PD-L1 expression on tumour-infiltrating immune cells covering ≥1% of the tumour area using the VENTANA PD-L1 (SP142) Assay.

The target sample size was approximately 2700 patients. To reduce selection bias, it was planned to enrol eligible patients at each site uniformly and consecutively. Additionally, to enrol a more homogeneous population, eligibility was restricted to patients treated with systemic therapy (i.e., not enrolling patients considered ineligible for systemic therapy because of frailty or very poor prognosis at one extreme, or less aggressive disease not requiring systemic therapy at the other extreme).

Medical/treatment history and outcomes data were retrospectively extracted from medical records. Treatment was selected according to local practice before enrolment into this non-interventional secondary data-use study; therefore, treatment choice was independent of participation in this retrospective study. There were no mandatory study visits or additional tests. If applicable per local regulations, written informed consent for study participation was required from all patients or a legally authorised representative. If consent was required according to local regulations but could not be obtained (e.g., from deceased patients) a waiver was required from the institutional review board/ethics committee. All patients’ privacy rights were observed. The study was conducted in full conformance with the Guidelines for Good Pharmacoepidemiology Practice, each site’s institutional review board and the laws and regulations of each participating country.

### 2.2 Study objectives

The primary objective was to determine the prevalence of PD-L1-positive status assessed in the local laboratory on primary and/or metastatic tissue. The secondary objective was to evaluate the inter-observer concordance between local and central PD-L1 testing. These objectives have been reported elsewhere [13]. Here we focus on prespecified exploratory clinical objectives, which included describing the baseline demographic and clinicopathologic characteristics among patients with eTNBC or mTNBC according to PD-L1 status, describing treatments administered in the neoadjuvant, adjuvant and metastatic settings, and evaluating clinical outcomes according to PD-L1 status (per central assessment), specifically: pathologic complete response (pCR) rate in the eTNBC cohort among patients treated with neoadjuvant chemotherapy; invasive disease-free survival (iDFS) in patients with eTNBC; progression-free survival (PFS) in patients with mTNBC who received first-line chemotherapy; and overall survival (OS) in patients with eTNBC and those with mTNBC. pCR was defined as the eradication of invasive disease in the breast and lymph nodes irrespective of ductal carcinoma in situ (ypT0/Tis ypN0). iDFS was defined as the interval between last surgery with curative intent before starting adjuvant chemotherapy until the earliest event of local or regional invasive recurrence, contralateral breast cancer recurrence, metastatic recurrence, breast or non-breast second cancer or death from any cause. PFS was defined as the interval between initiation of first-line chemotherapy and first documented disease progression or death, whichever occurred earlier. OS (evaluated separately in the eTNBC and mTNBC cohorts) was defined as the interval between confirmed histopathological diagnosis and death from any cause.

### 2.3 Statistical analysis

Exploratory endpoints were summarised using descriptive statistics. Kaplan–Meier methodology was used to estimate iDFS, PFS and OS. Kaplan–Meier curves should be interpreted with caution: any comparisons between groups are purely descriptive and exploratory, with important limitations (including the retrospective setting, non-randomised design and missing data). Annual survival rates are presented together with 95% confidence intervals (CIs) derived from Greenwood’s formula, to convey the precision of these estimates. Patients with no documented chemotherapy start date were excluded from PFS analyses. In patients with only partially known dates (i.e., missing day or month) a conservative approach was adopted, with partially known start dates imputed to the latest potential date (i.e., last day of the month if the day was missing, 31 December if only the year was known) and partially known event dates imputed to the earliest potential date (i.e., first day of the month if only the day was missing, 1 January if only the year was known). If this conservative imputation resulted in a negative time to event (e.g., recorded recurrence date before surgery date), the time to event was imputed as 1 day.

## 3 Results

**Fig. 1:**
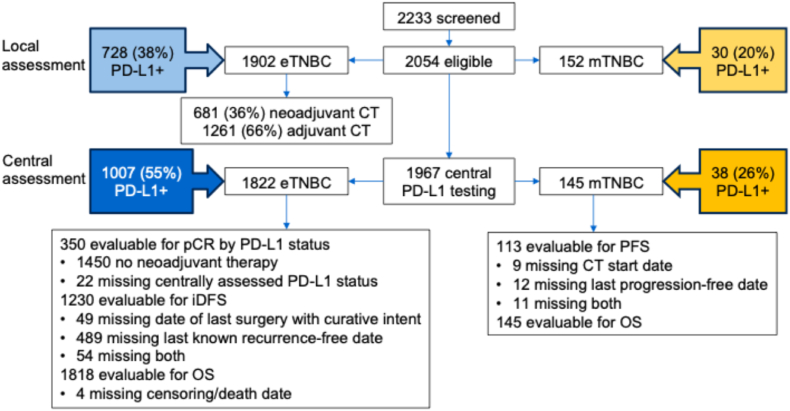
Analysis populations and PD-L1-positive prevalence. CT, chemotherapy; eTNBC, early-stage triple-negative breast cancer; iDFS, invasive disease-free survival; mTNBC, metastatic triple-negative breast cancer; OS, overall survival; pCR, pathologic complete response; PD-L1, programmed death-ligand 1; PFS, progression-free survival.

Overall, 2054 eligible patients were enrolled from 39 sites in 19 countries: 1902 with eTNBC and 152 with mTNBC (Fig. 1, Appendix Table A.1, Appendix Table A. 2). All samples were tested locally for PD-L1 status; central laboratory testing was available for 1967 samples (96%).

### 3.1 eTNBC cohort

In the eTNBC cohort, the prevalence of PD-L1-positive status was 38% (95% CI: 36–41%) in 1902 patients assessed locally and 55% in 1822 patients with available PD-L1 status according to central laboratory testing. Demographic and disease characteristics were broadly similar in the subgroups with PD-L1-positive versus PD-L1-negative tumours by central assessment. However, compared with the PD-L1-negative population, the PD-L1-positive population included a slightly higher proportion of patients self-reporting as White (44% vs 37%) and a higher proportion with tumour characteristics typical of lower-risk disease (node negative, stage I/II, T1/2) (Table 1). *BRCA* mutation status was unknown in most patients.

**Table 1:** Patient characteristics in the eTNBC cohort according to PD-L1 status by central assessment.

Characteristic	PD-L1-positive (n = 1007)	PD-L1-negative (n = 815)
Age, years, median (range)	52 (21–93)	52 (25–98)
Race, n (%)		
American Indian/Alaska Native	43 (4)	54 (7)
Asian	224 (22)	181 (22)
Black/African American	47 (5)	39 (5)
Native Hawaiian/other Pacific Islander	2 (<1)	0
White	441 (44)	300 (37)
Other^a^	106 (11)	110 (13)
Missing/unknown	144 (14)	131 (16)
ECOG PS, n (%)		
0	659 (65)	511 (63)
1	235 (23)	197 (24)
2	15 (1)	18 (2)
Missing	98 (10)	89 (11)
Stage at diagnosis, n (%)		
I	150 (15)	90 (11)
II	413 (41)	312 (38)
III	262 (26)	265 (33)
Missing	182 (18)	148 (18)
Nodal status, n (%)		
N0	494 (49)	333 (41)
N1	284 (28)	236 (29)
N2	88 (9)	100 (12)
N3	58 (6)	53 (7)
Nx/missing	83 (8)	93 (11)
Tumour category, n (%)		
T1	241 (24)	142 (17)
T2	499 (50)	352 (43)
T3	112 (11)	116 (14)
T4	81 (8)	128 (16)
Tx/missing	72 (7)	75 (9)
T0/Tis	2 (<1)	2 (<1)
Histological grade, n (%)		
1	2 (<1)	14 (2)
2	212 (21)	267 (33)
3	740 (73)	485 (60)
Missing	53 (5)	49 (6)
Histology^b^, n (%)		
Invasive ductal carcinoma	837 (83)	647 (79)
Medullary carcinoma	37 (4)	7 (1)
Invasive lobular carcinoma	14 (1)	28 (3)
Invasive papillary carcinoma	3 (<1)	10 (1)
Mucinous carcinoma	0	2 (<1)
Mixed pathology	10 (1)	3 (<1)
Other	105 (10)	107 (13)
Missing	18 (2)	24 (3)
*BRCA* status, n (%)		
Wildtype	79 (8)	50 (6)
Mutated	68 (7)	34 (4)
Unknown/not assessed	860 (85)	731 (90)
Mutated among those with known status	68/147 (46)	34/84 (40)

Abbreviations: ECOG PS, Eastern Cooperative Oncology Group performance status; eTNBC, early-stage triple-negative breast cancer; PD-L1, programmed death-ligand 1.

a Includes Arab (Middle Eastern), Arabic, East Africa, Indian, Mestizo, Middle Eastern.

b More than once answer possible.

Among all 1902 patients with eTNBC, 681 (36%) received neoadjuvant treatment. In most cases, neoadjuvant chemotherapy included an anthracycline and/or a taxane (Table 2). Only 12% received a platinum-based neoadjuvant chemotherapy regimen. Likewise, most patients receiving adjuvant chemotherapy were treated with an anthracycline- and taxane-containing regimen, and few received platinum. Surgical details are shown in Appendix Table A.3. Most patients completed the planned treatment.

**Table 2:** Treatment patterns in the eTNBC cohort.

Treatment for eTNBC, n (%)	Neoadjuvant (n = 681)	Adjuvant (n = 1261)
Chemotherapy regimen^a^		
Anthracycline	196 (29)	324 (26)
Taxane	135 (20)	295 (23)
Anthracycline and taxane	395 (58)	639 (51)
Platinum	83 (12)	58 (5)
Other	119 (17)	264 (21)
Premature discontinuation	85 (12)	100 (8)
Progression	35 (5)	25 (2)
Toxicity	24 (4)	43 (3)
Patient or physician decision	8 (1)	20 (2)
Other	18 (3)	12 (1)

Abbreviation: eTNBC, early-stage triple-negative breast cancer.

a More than one answer possible.

pCR status was available in 372 (55%) of the 681 patients who received neoadjuvant chemotherapy; both pCR status and PD-L1 status were available in 350 (51%) of these patients. There was no meaningful numerical difference in pCR rates between patients with PD-L1-positive tumours (27/168; 16%, 95% CI: 11–23%) and those with PD-L1-negative tumours (31/182; 17%, 95% CI: 12–23%). Univariable logistic regression analyses of the 372 patients regardless of PD-L1 status revealed no predictive effect of stage at initial diagnosis, histological grade at diagnosis or type of neoadjuvant regimen (anthracycline, taxane, anthracycline and taxane, platinum, other).

**Fig. 2:**
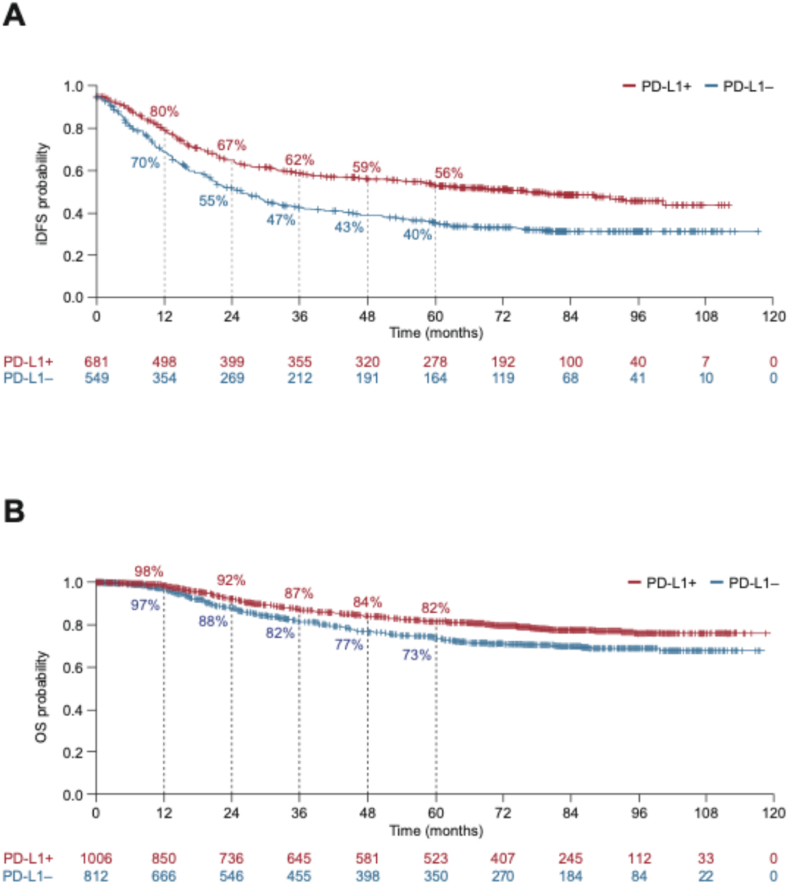
Clinical outcomes in the eTNBC cohort with centrally assessed PD-L1 status. A) iDFS (n = 1230); B) OS (n = 1818). Median values are not shown with so few patients at risk and censoring in a large proportion of patients. eTNBC, early-stage triple-negative breast cancer; iDFS, invasive disease-free survival; OS, overall survival; PD-L1, programmed death-ligand 1.

In 592 patients, iDFS was not evaluable because of missing start and/or censoring dates (49 without date of last surgery with curative intent [start], 489 without last date known without recurrence recorded [censoring] and 54 missing both). Among the 1230 patients with centrally assessed PD-L1 status who were evaluable for iDFS, 639 (52%; 44% in the PD-L1-positive subgroup and 61% in the PD-L1-negative subgroup) had experienced an iDFS event. iDFS appeared more favourable in patients with PD-L1-positive eTNBC (n = 681) than PD-L1-negative eTNBC (n = 549) with increasing separation of the Kaplan–Meier curves over time (Fig. 2A). The 5-year iDFS rates were 56% (95% CI: 52–60%) in the PD-L1-positive subgroup and 40% (95% CI: 36–44%) in the PD-L1-negative subgroup.

All but four patients were evaluable for OS. Deaths had been recorded in 348 patients (19%; 17% in the PD-L1-positive subgroup and 22% in the PD-L1-negative subgroup). Similar to iDFS, annual OS rates appeared consistently higher in patients with PD-L1-positive versus PD-L1-negative eTNBC, with increasing divergence over time (Fig. 2B). At 5 years, OS rates were 82% (95% CI: 79–84%) in the PD-L1-positive subgroup and 73% (95% CI: 70–77%) in the PD-L1-negative subgroup.

### 3.2 mTNBC cohort

In the cohort of 152 patients with mTNBC, the prevalence of PD-L1-positive status was 20% (95% CI: 14–27%) by local assessment and 26% in the 145 patients with centrally assessed PD-L1 status. Among these 145 patients, most (83%) had de novo mTNBC. Compared with the PD-L1-negative subgroup, the PD-L1-positive subgroup was slightly older and included a higher proportion of patients self-reporting as White (58% vs 48%) and with Eastern Cooperative Oncology Group (ECOG) performance status of 0 (50% vs 41%), and a lower proportion with liver metastases (13% vs 33%), lung metastases (37% vs 54%) and self-reporting as Asian (5% vs 19%) (Table 3).

**Table 3:** Patient characteristics in the mTNBC cohort according to PD-L1 status by central assessment (n = 145).

Characteristic	All mTNBC		De novo mTNBC subset	
	PD-L1-positive (n = 38)	PD-L1-negative (n = 107)	PD-L1-positive (n = 30)	PD-L1-negative (n = 90)
Age, years, median (range)	61 (31–82)	56 (24–89)	NA	NA
Race, n (%)				
American Indian/Alaska Native	0	1 (1)	0	NA
Asian	2 (5)	20 (19)	NA	NA
Black/African American	0	6 (6)	0	NA
White	22 (58)	51 (48)	NA	NA
Other^a^	4 (11)	14 (13)	NA	NA
Missing/unknown	10 (26)	15 (14)	NA	NA
ECOG PS, n (%)				
0	19 (50)	44 (41)	15 (50)	32 (36)
1	10 (26)	34 (32)	7 (23)	31 (34)
2	5 (13)	13 (12)	5 (17)	12 (13)
Missing	4 (11)	16 (15)	3 (10)	15 (17)
De novo mTNBC, n (%)	30 (79)	90 (84)	30 (100)	90 (100)
Nodal status at diagnosis, n (%)				
N0	7 (18)	13 (12)	3 (10)	5 (6)
N1	9 (24)	21 (20)	6 (20)	16 (18)
N2	9 (24)	19 (18)	9 (30)	19 (21)
N3	5 (13)	19 (18)	5 (17)	17 (19)
Nx/missing	8 (21)	35 (33)	7 (23)	33 (37)
Tumour category at diagnosis, n (%)				
T1	2 (5)	8 (7)	0	4 (4)
T2	11 (29)	19 (18)	7 (23)	15 (17)
T3	5 (13)	12 (11)	4 (13)	7 (8)
T4	14 (37)	36 (34)	14 (47)	34 (38)
Tx/missing	6 (16)	32 (30)	5 (17)	30 (33)
Histological grade at initial diagnosis, n (%)				
1	1 (3)	1 (1)	0	0
2	5 (13)	32 (30)	5 (17)	27 (30)
3	21 (55)	60 (56)	15 (50)	49 (54)
Missing	11 (29)	14 (13)	10 (33)	14 (16)
Metastatic site, n (%)				
Liver	5 (13)	35 (33)	5 (17)	31 (34)
Lung	14 (37)	58 (54)	12 (40)	54 (60)
Brain	7 (18)	12 (11)	4 (13)	10 (11)
Lymph nodes	20 (53)	39 (36)	16 (53)	33 (37)
Bone	10 (26)	31 (29)	10 (33)	26 (29)

Abbreviations: ECOG PS, Eastern Cooperative Oncology Group performance status; mTNBC, metastatic triple-negative breast cancer; NA, not available; PD-L1, programmed death-ligand 1.

a Includes Arabic (n = 13), Middle Eastern (n = 4) and Mestizo (n = 1).

The most frequently used therapies for mTNBC were anthracycline and/or taxane in the first-line setting, taxane, capecitabine and/or platinum in the second-line setting and capecitabine or platinum in the third-line setting (Table 4). Seven patients received bevacizumab (four as first-line therapy and three as second-line therapy; six in combination with paclitaxel and one with capecitabine).

**Table 4:** Treatment patterns in the mTNBC cohort.

Treatment	No. of patients (%)
Chemotherapy (n = 152)	
Yes	132 (87)
Missing	20 (13)
Number of lines of chemotherapy (n = 152)	
1	54 (36)
2	42 (28)
3	36 (24)
Missing	20 (13)
Most common first-line chemotherapy regimens (n = 132)^a^	
Anthracycline	63 (48)
Taxane	59 (45)
Platinum	13 (10)
Reason for discontinuing first-line chemotherapy (n = 132)	
Disease progression	68 (52)
Death	5 (4)
Other^b^	38 (29)
Not applicable	21 (16)
Most common second-line chemotherapy regimens (n = 78)^a^	
Taxane	36 (46)
Capecitabine	18 (23)
Platinum	17 (22)
Gemcitabine	12 (15)
Reason for discontinuing second-line chemotherapy (n = 78)	
Disease progression	44 (56)
Death	7 (9)
Other^c^	19 (24)
Not applicable	8 (10)
Most common third-line chemotherapy regimens (n = 36)^a^	
Capecitabine	9 (25)
Platinum	8 (22)
Taxane	6 (17)
Gemcitabine	6 (17)
Vinorelbine	4 (11)
Eribulin	4 (11)
Reason for discontinuing third-line chemotherapy (n = 36)	
Disease progression	23 (64)
Death	4 (11)
Other^d^	8 (22)
Not applicable	1 (3)
Palliative locoregional therapy (n = 152)^a^	
Yes	52 (34)
Radiotherapy	40 (26)
Surgery	23 (15)

Abbreviations: ECOG PS, Eastern Cooperative Oncology Group performance status; mTNBC, metastatic triple-negative breast cancer.

a More than one answer possible.

b Unknown (n = 8), physician decision (n = 5), toxicity/cardiac issues/lung damage (n = 9), completed planned cycles (n = 2), surgery (n = 2), complete/partial response (n = 2), stable disease, (n = 1), partial response/anthracycline toxicity (n = 1), cumulative anthracycline dose (n = 1), patient decision (n = 3), ECOG PS 3 (n = 1), lost to follow-up (n = 3).

c Worsened/poor performance status (n = 4), patient decision (n = 1), complete response (n = 1), stable disease (n = 1), surgery (n = 1), toxicity (n = 4), lost to follow-up (n = 3), unspecified/unknown (n = 4).

d Toxicity (n = 2), worsening condition (n = 2), planned radiation (n = 1), unspecified/unknown (n = 2), lost to follow-up (n = 1).

**Fig. 3:**
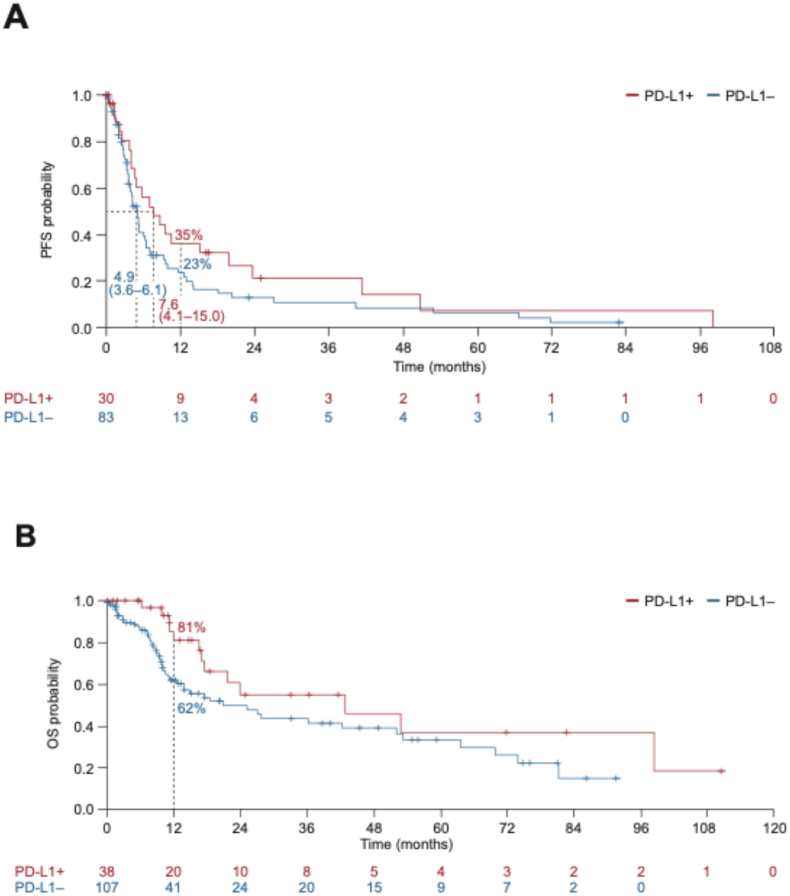
Clinical outcomes in the mTNBC cohort with centrally assessed PD-L1 status. A) PFS (n = 113); B) OS (n = 145). mTNBC, metastatic triple-negative breast cancer; OS, overall survival; PD-L1, programmed death-ligand 1; PFS, progression-free survival.

PFS could not be estimated in 32 patients because of missing chemotherapy start date in nine patients, missing last known progression-free date in 12 patients and missing both (start and censoring dates) in 11 patients. In the 113 patients evaluable for PFS, 95 (84%) had experienced disease progression or death. Numerically longer median PFS was observed in patients with PD-L1-positive mTNBC (median 7.6 months; 95% CI: 4.1–15.0 months) than PD-L1-negative mTNBC (median 4.9 months; 95% CI: 3.6–6.1 months). The 1-year PFS rates were 35% (95% CI: 18–53%) and 23% (95% CI: 13–34%), respectively (Fig. 3A). The number of patients at risk beyond 1 year was too small for meaningful interpretation. A similar pattern was observed for OS (Fig. 3B), with median OS of 42.9 months (95% CI: 17.1–not evaluable) in patients with PD-L1-positive mTNBC and 20.8 months (95% CI: 12.6–51.9 months) in PD-L1-negative mTNBC. However, with so few patients and events, particularly in the PD-L1-positive subgroup, median values are unreliable.

In post hoc subgroup analyses, median OS was 23.9 months (95% CI: 17.1–not estimable) in 30 patients with PD-L1-positive de novo mTNBC and 20.8 months (95% CI: 11.2–53.2) in 90 patients with PD-L1-negative de novo mTNBC (Appendix Fig. A.1). The 1-year OS rates were 82% (95% CI: 59–93%) and 62% (95% CI: 49–72%), respectively. The subgroup with recurrent TNBC was too small for meaningful interpretation (Appendix Fig. A.1).

## 4 Discussion

The VANESSA dataset provides information on treatment patterns, outcomes and the prognostic impact of PD-L1 status in one of the largest reported international real-world populations of patients with TNBC. Most were diagnosed with eTNBC, for which there is limited global information [[17], [18], [19]], and all were treated before immunotherapy was adopted into the routine clinical management of TNBC.

In the eTNBC cohort, anthracycline- and taxane-containing regimens were widely used, usually as adjuvant therapy, reflecting typical clinical practice in 2014–2017. Five-year OS rates were 82% in patients with PD-L1-positive eTNBC and 73% in those with PD-L1-negative eTNBC. These outcomes are in line with expectations at the time. Nowadays, chemotherapy is generally offered before surgery and neoadjuvant chemotherapy with pembrolizumab is established for patients with higher risk eTNBC [3,4,20]. Advances in diagnosis and local management, the refinement of chemotherapy regimens for eTNBC (e.g., adoption of dose-dense regimens, inclusion of platinum and pembrolizumab) and improved treatment for metastatic disease (e.g., immune checkpoint inhibitors [5,7,8], PARP inhibitors [[21], [22], [23], [24]] and antibody–drug conjugates [25]) have contributed to an improved prognosis since the VANESSA data collection period.

In the eTNBC cohort, the pCR rates of 16–17% are much lower than pCR rates of up to 57% with modern chemotherapy regimens in contemporary trials [1,2,26,27], with the important caveat that pCR rates in VANESSA are calculated based on less than half of the patients receiving neoadjuvant chemotherapy because of missing data, and among those patients, the lack of recorded pCR in medical records may not represent absence of pCR. The heterogeneity of the VANESSA population makes it difficult to select a reasonable historical benchmark for comparisons; however, global variations in healthcare systems, treatment strategies and access to therapies and the evolving treatment landscape may contribute to apparent differences.

It is more difficult to interpret treatment patterns, the prevalence of PD-L1-positive status and clinical outcomes in the mTNBC cohort because of the unexpectedly small sample size and the exceptionally high proportion of patients with de novo mTNBC (83% versus approximately 30% in randomised phase III trials and other real-world datasets [7,28,29]). The predominance of de novo mTNBC may reflect detection patterns in the participating countries, selection bias towards newly diagnosed patients (early stage and de novo) versus patients with relapsed/recurrent TNBC during the enrolment period, or deviation from the planned consecutive enrolment because of the unavailability of samples and/or medical records in patients with an earlier diagnosis of mTNBC, inability to contact patients and/or local regulations for informed consent signatures. In this predominantly chemonaïve cohort, only 45% of patients received a first-line taxane and 48% an anthracycline; platinum and capecitabine use was higher in later lines. Median PFS (7.6 months in PD-L1-positive mTNBC, 4.9 months in PD-L1-negative mTNBC) was consistent with published data from a similar period (median 5.4 months with first-line chemotherapy alone [30]), although median PFS in the PD-L1-positive subgroup was longer than reported with chemotherapy alone in the PD-L1-positive populations of the KEYNOTE-355, IMpassion130 and IMpassion131 trials (5–6 months) [5,7,28]. This may reflect the high percentage with de novo mTNBC associated with more favourable tumour biology and clinical outcomes [29]. Spatiotemporal variations in PD-L1 expression and/or diagnostic differences may also contribute, as described elsewhere [13].

In both eTNBC and mTNBC, PD-L1-positive status by SP142 was associated with more favourable long-term outcomes, similar to reports from smaller studies [31,32] and possibly explained by tumour-intrinsic characteristics and/or the host immune response. The higher pCR rates and lower event rates in PD-L1-positive than PD-L1-negative subgroups of randomised clinical trials in eTNBC also support a prognostic effect of PD-L1 status [1,4], although we observed no such difference in pCR rates according to PD-L1 status in VANESSA. Whether these observations reflect independent prognostic value or association with clinical characteristics of more indolent disease is difficult to ascertain, but the results add to accumulating data suggesting better clinical outcomes in patients with PD-L1-positive TNBC.

A limitation of these analyses is the small size of the mTNBC cohort and the applicability of findings from a predominantly de novo mTNBC population. Another challenge limiting interpretation of long-term outcomes is missing data: data in routine practice are typically less complete than in prospective clinical trials with time-to-event primary outcomes. Furthermore, data completeness varies considerably between countries and centres. Incomplete details of chemotherapy dosing and schedules and progression or recurrence dates are common in secondary data-use studies, requiring exclusion of patients from the analyses or imputation of missing dates. In the eTNBC cohort, iDFS could not be calculated in one-third of patients because of missing iDFS start and/or end dates. Similarly, chemotherapy start dates or other details were missing in >20% of the mTNBC cohort. In the remaining patients, conservative imputation of missing dates potentially led to underestimation of iDFS and PFS and, particularly in the poor-prognosis mTNBC setting, such artificial shortening by up to 1 year has a substantial impact on the reliability of estimates. In both cohorts OS was estimated from the date of histological diagnosis, which allowed inclusion of all patients irrespective of data completeness, and potentially artificially lengthened OS estimates compared with randomised clinical trials (where OS is typically estimated from randomisation) or many other real-world or single-arm studies (where OS is estimated from treatment initiation). Furthermore, OS may be underestimated in secondary data-use studies as patients with terminal cancer choosing to discontinue hospital visits may be recorded as lost to follow-up.

Another consideration is the data collection period (2014–2017), which provided long-term clinical outcome data, but introduces complexity in rapidly evolving therapeutic fields such as TNBC. The treatment landscape in both eTNBC and mTNBC has been transformed from a chemotherapy-alone approach (with bevacizumab for mTNBC in some healthcare settings but not routinely including platinum) to the current wide array of therapies offering clinically and/or statistically significant improvements in OS, including targeted treatment options (depending on detection of biomarkers) and antibody–drug conjugates. The changing landscape has also revealed new markers with prognostic value, such as *BRCA1/2* pathogenic variants, which were not routinely assessed before 2018 but have become increasingly important in the work-up for eTNBC when evaluating risk factors and selecting suitability for PARP inhibitor therapy. Finally, the varied quality of available information on treatment administration in a retrospective study affects analysis and interpretation of treatment patterns.

In conclusion, although results in mTNBC are difficult to contextualise, clinical data from almost 2000 patients with eTNBC provide an interesting snapshot of treatment patterns and outcomes in a global real-world population spanning four continents. These data may be valuable in benchmarking for future clinical trials in eTNBC.

## CRediT authorship contribution statement

**Lazar Popovic:** Writing – review & editing, Writing – original draft, Resources. **Romualdo Barroso-Sousa:** Writing – review & editing. **Nagi S. El Saghir:** Writing – review & editing, Resources. **Rebecca Dent:** Writing – review & editing. **Sitki Tuzlali:** Writing – review & editing. **Saad Akhtar:** Writing – review & editing, Resources. **Elona Juozaityté:** Writing – review & editing, Resources. **Janis Eglitis:** Writing – review & editing, Resources. **Dinesh C. Doval:** Writing – review & editing, Resources. **Carlos A. Castaneda:** Writing – review & editing, Resources. **Alisan Zirtiloglu:** Writing – review & editing, Data curation. **Götz Hartleben:** Writing – review & editing, Methodology, Conceptualization. **Regula Deurloo:** Writing – review & editing, Methodology, Conceptualization. **Paula Toro:** Writing – review & editing, Conceptualization. **Iman Estaytieh:** Writing – review & editing, Data curation, Conceptualization. **Enya Weber:** Writing – review & editing, Writing – original draft, Methodology, Formal analysis. **João Mouta:** Writing – review & editing, Writing – original draft, Supervision, Conceptualization. **Corrado D’Arrigo:** Conceptualization, Resources, Writing – review & editing.

## Data availability statement

Data from which the published results of the VANESSA study are derived are archived by the study sponsor, and can be made publicly available upon request. Requests for the data underlying this publication require a detailed, hypothesis-driven statistical analysis plan that is collaboratively developed by the requestor and company subject matter experts. Direct such requests to joao.mouta@roche.com for consideration.

## Ethics statement

If applicable per local regulations, written informed consent for study participation was required from all patients or a legally authorised representative. If consent was required according to local regulations but could not be obtained (e.g., from deceased patients) a waiver was required from the institutional review board/ethics committee. All patients’ privacy rights were observed. The study was conducted in full conformance with the Guidelines for Good Pharmacoepidemiology Practice, each site’s institutional review board and the laws and regulations of each participating country.

## Funding source

This study was sponsored by F. Hoffmann-La Roche Ltd. The sponsor was involved in the design of the study, collection, analysis and interpretation of the data and report writing. The first author had the final decision in submitting the article for publication.

## Declaration of competing interest

LP reports speaker/advisor/investigator roles for AstraZeneca, MSD, BMS, Pfizer, Roche, Merck, Novartis, Lilly, Gilead, Takeda, Helsinn, Astellas, Janssen, Sanofi, Sandoz, Actavis, Amgen, Archigen, Amicus, Taiho, Infinity, Bioclin, G1 Therapeutics, MEI Pharma, Immunocore/Medison, NAPO Pharmaceuticals, Oktal, PharmaSwiss, AbbVie, Medica Linea, MAK Pharma, Agendia, Recordati, Incyte and Bicycle Therapeutics. R B-S reports receiving speaker bureau fees from AstraZeneca, Daiichi Sankyo, Eli Lilly, Pfizer, Novartis, Merck and Roche, has served as a consultant/advisor for AstraZeneca, Eli Lilly, Libbs, Roche and Merck, has received support for attending medical conferences from AstraZeneca, Roche, Eli Lilly, Daiichi Sankyo and Merck, has received institutional research funding from AstraZeneca and Daiichi Sankyo and owns stocks in the educational company RD Educação Médica LTDA. NSES reports honoraria for lectures from AstraZeneca, Novartis and Roche. RD reports consulting fees/honoraria/advisory boards/travel from AstraZeneca, MSD, Pfizer, Eisai, Novartis, Daiichi Sankyo, Roche, Eli Lilly & Company, Genentech and Gilead and research grants from AstraZeneca and Roche. SA reports financial support from F. Hoffmann-La Roche. AZ, GH, RD, PT, IE, EW and JM are current or former employees of and shareholders in Roche. CD’A is the founder of the Poundbury Cancer Institute and has received personal funds for consultation and advisory roles as well as funds for research projects and studies from Roche, AstraZeneca, Daiichi Sankyo Inc., Merck Sharp & Dohme and Pfizer. All authors received non-financial support in the form of statistical analysis (Excelya) and medical writing (Medi-Kelsey Ltd), funded by F. Hoffmann-La Roche Ltd. ST and JE declare no other conflicts of interest.
