# Supplementary material for: Treatment patterns and clinical outcomes according to PD-L1 status in >2000 patients with early-stage or metastatic triple-negative breast cancer treated in the real-world setting: VANESSA study results
Source: Breast. 2026 Feb 5;86:104720. doi: 10.1016/j.breast.2026.104720 (PMC12914666; doi:10.1016/j.breast.2026.104720)
Supplement: Multimedia component 1 [file mmc1.docx]

**Treatment patterns and clinical outcomes according to PD-L1 status in >2000 patients with early-stage or metastatic triple-negative breast cancer treated in the real-world setting: VANESSA study results**

**Appendix Table A.1.**

Number of eligible patients by country and year of diagnosis (n = 2054).

| No. of patients | 2014 (n = 489) | 2015 (n = 521) | 2016 (n = 537) | 2017 (n = 506) |
| --- | --- | --- | --- | --- |
| Algeria (n = 99) | 24 | 26 | 26 | 23 |
| Chile (n = 56) | 15 | 16 | 14 | 11 |
| Finland (n = 40) | 10 | 10 | 10 | 10 |
| Germany (n = 86)^a^ | 25 | 26 | 16 | 18 |
| India (n = 200) | 50 | 51 | 50 | 49 |
| Italy (n = 150) | 29 | 44 | 35 | 42 |
| Kenya (n = 80) | 19 | 20 | 22 | 19 |
| Latvia (n = 54) | 15 | 14 | 14 | 11 |
| Lebanon (n = 128) | 25 | 25 | 44 | 34 |
| Lithuania (n = 117) | 29 | 32 | 26 | 30 |
| Peru (n = 100) | 25 | 25 | 25 | 25 |
| Republic of Korea (n = 120) | 30 | 30 | 30 | 30 |
| Saudi Arabia (n = 139) | 33 | 36 | 36 | 34 |
| Serbia (n = 268) | 56 | 63 | 78 | 71 |
| South Africa (n = 37) | 10 | 8 | 8 | 11 |
| Tunisia (n = 117) | 29 | 29 | 29 | 30 |
| Türkiye (n = 99) | 24 | 23 | 31 | 21 |
| United Kingdom (n = 44) | 11 | 14 | 13 | 6 |
| Vietnam (n = 120) | 30 | 29 | 30 | 31 |

Abbreviation: eTNBC, early triple-negative breast cancer.

^a^ Year of diagnosis missing in one patient (breast-conserving surgery for eTNBC in 2017).

**Appendix Table A.2.** Participating sites and principal physicians. Three sites enrolled patients subsequently excluded from the eligible patient population.

| Country | Principal physician | Site |
| --- | --- | --- |
| Serbia (*n =* 268) | Nataša Medić-Milijić | Institute of Oncology and Radiology of Serbia, Belgrade |
|  | Ana Cvetanovic | University Clinical Centre Nis, Nis |
|  | Lazar Popovic | Oncology Institute of Vojvodina, Sremska Kamenica |
| India (*n =* 200) | S. Ganapathi Ramanan | Apollo Hospitals, Anna Salai, Chennai |
|  | Pavithran Keechilat | Amrita Institute of Medical Sciences, Ponekkara |
|  | Dinesh Doval | Rajiv Gandhi Cancer Institute & Research Center, Rohini, New Delhi |
|  | Sudeep Gupta | Tata Memorial Hospital, Parel, Mumbai |
| Italy (*n =* 150) | Filippo Giovanardi | AUSL-IRCCS di Reggio Emilia, Ospedale Civile di Guastalla, Reggio Emilia |
|  | Giulia Bianchi | IRCCS Istituto Nazionale dei Tumori, Milano |
|  | Patrizia Vici | IRCCS Istituto Regina Elena, Roma |
|  | Claudia Bighin | IRCCS A.O.U San Martino – IST, Genova |
| Saudi Arabia (*n =* 139) | Abdulmohsen Alkushi | National Guard King Abdulaziz Medical City, Riyadh |
|  | Sayed Akhtar | King Faisal Specialist Hospital & Research Centre, Riyadh |
| Lebanon (*n =* 128) | Nagi El Saghir | American University of Beirut – Medical Center, Beirut |
|  | Fadi Farhat | Hammoud Hospital, Saida |
|  | David Attalah | Hotel Dieu de France, Beirut |
| Republic of Korea (*n =* 120) | Gyungyub Gong | Asan Medical Center, Seoul |
| Vietnam (*n =* 120) | Tu Thai Anh | Ho Chi Minh City Oncology Hospital, Ho Chi Minh |
|  | To Ta | K Hospital, Ha Noi |
| Lithuania (*n =* 117) | Elona Juozaityte | Hospital of Lithuanian University of Health Sciences Kaunas Clinics, Kaunas |
|  | Monika Drobniene | National Cancer Institute, Vilnius |
| Tunisia (*n =* 117) | Maha Driss | Salah Azaiez Institute, Tunis |
|  | Moncef Mokni | Farhat Hached Hospital, Sousse |
| Peru (*n =* 100) | Henry Gomez-Moreno | Oncosalud Sac, Lima |
|  | Carlos Castañeda | Instituto Nacional de Enfermedades Neoplasicas, Lima |
| Algeria (*n =* 99) | Amel Ladjeroud and Mohammed Oukkal | CPMC, Alger, Algiers (satellite site: CHU Béni-Messous, Beau-Fraisiers) |
|  | Hanene Djedi | CHU Annaba, Hôpital Dorban, Annaba |
| Türkiye (*n =* 99) | Handan Kaya | Marmara University Faculty of Medicine, Istanbul |
|  | Arsenal Alikanoglu | Antalya Research and Training Hospital, Antalya |
|  | Ebru Tastekin | Trakya University Medical Faculty, Edirne |
|  | Melek Ergin | Cukurova University Medical Faculty, Adana |
| Germany (*n =* 86) | Ingolf Juhasz-Böss | Universitätsklinikum Freiburg, Freiburg |
|  | Eugen Ruckhäberle | Universitätsklinikum Düsseldorf, Düsseldorf |
| Kenya (*n =* 80) | Shahin Sayed | Aga Khan University Hospital, Nairobi |
| Chile (*n =* 56) | Felipe Reyes | Fundacion Arturo Lopez Perez, Santiago |
|  | Cesar Sanchez | Centro de Cancer Pontificie Universidad Catolica de Chile, Santiago |
| Latvia (*n =* 54) | Janis Eglitis | Riga East Clinical University Hospital Latvian Oncology Centre, Riga |
| UK (*n =* 44) | Corrado D’Arrigo | Poundbury Cancer Institute, Dorchester |
| Finland (*n =* 40) | Antti Ellonen | Turku University Central Hospital, Turku |
| South Africa (*n =* 37) | Georgia Savva Demetriou | Charlotte Maxeke Johannesburg Academic Hospital, Johannesburg |
| Morocco (*n =* 0) | Nawfel Mellas | Centre Hospitalier Universitaire Hassan II |
|  | Hassan Errihani | Institut National d’Oncologie Sidi Mohammed Ben Abdellah |

**Appendix Table A.3.**

Details of surgery in the eTNBC cohort (n = 1902).

| Type of surgery | No. of patients (%) |
| --- | --- |
| Breast surgery |  |
| None | 101 (5) |
| Breast-conserving surgery | 701 (37) |
| Simple mastectomy | 295 (16) |
| Modified radical mastectomy | 717 (38) |
| Re-excision of surgical margins | 7 (<1) |
| Other | 66 (3) |
| Missing (not collected) | 15 (1) |
| Axillary surgery |  |
| None | 278 (15) |
| Sentinel lymph node biopsy alone | 473 (25) |
| Axillary sampling | 64 (3) |
| Axillary dissection following sentinel lymph node biopsy | 194 (10) |
| Axillary dissection | 833 (44) |
| Other | 44 (2) |
| Missing (not collected) | 16 (1) |

Abbreviation: eTNBC, early-stage triple-negative breast cancer.

**Appendix Fig. A.1.** OS by PD-L1 status. A) Subgroup of patients with de novo mTNBC (n = 120). B) Subgroup of patients with recurrent mTNBC. mTNBC, metastatic triple-negative breast cancer; OS, overall survival; PD-L1, programmed death-ligand 1.
